# Integrating metabolomics and network pharmacology to assess the effects of quercetin on lung inflammatory injury induced by human respiratory syncytial virus

**DOI:** 10.1038/s41598-023-35272-8

**Published:** 2023-05-17

**Authors:** Ya-Lei Sun, Pei-Pei Zhao, Cheng-Bi Zhu, Ming-Chen Jiang, Xin-Min Li, Jia-Lei Tao, Chan-Chan Hu, Bin Yuan

**Affiliations:** 1 Department of Pediatrics, Affiliated Hospital of Nanjing University of Chinese Medicine, Nanjing, China; 2 Jiangsu Key Laboratory of Pediatric Respiratory Disease, Institute of Pediatrics, Affiliated Hospital of Nanjing University of Chinese Medicine, Nanjing, China; 3 Nanjing University of Chinese Medicine, Nanjing, China; 4 Henan University of Chinese Medicine, Zhengzhou, China

**Keywords:** Metabolic engineering, Respiratory tract diseases

## Abstract

Quercetin (QR) has significant anti-respiratory syncytial virus (RSV) effects. However, its therapeutic mechanism has not been thoroughly explored. In this study, a lung inflammatory injury model caused by RSV was established in mice. Untargeted lung tissue metabolomics was used to identify differential metabolites and metabolic pathways. Network pharmacology was used to predict potential therapeutic targets of QR and analyze biological functions and pathways modulated by QR. By overlapping the results of the metabolomics and the network pharmacology analyses, the common targets of QR that were likely to be involved in the amelioration of RSV-induced lung inflammatory injury by QR were identified. Metabolomics analysis identified 52 differential metabolites and 244 corresponding targets, while network pharmacology analysis identified 126 potential targets of QR. By intersecting these 244 targets with the 126 targets, hypoxanthine–guanine phosphoribosyltransferase (HPRT1), thymidine phosphorylase (TYMP), lactoperoxidase (LPO), myeloperoxidase (MPO), and cytochrome P450 19A1 (CYP19A1) were identified as the common targets. The key targets, HPRT1, TYMP, LPO, and MPO, were components of purine metabolic pathways. The present study demonstrated that QR effectively ameliorated RSV-induced lung inflammatory injury in the established mouse model. Combining metabolomics and network pharmacology showed that the anti-RSV effect of QR was closely associated with purine metabolism pathways.

The human respiratory syncytial virus (RSV) is an enveloped, negative-sense, single-stranded RNA (ssRNA) virus of the Pneumoviridae family. The genome is encapsulated in the nucleocapsid surrounded by a lipoprotein envelope, and the viral RNA encodes 10 subgenomic mRNAs and 11 proteins^1^. The major surface glycoprotein (G) facilitates virus attachment, and the fusion (F) protein mediates virus-cell fusion^2^. RSV is a leading cause of RSV pneumonia (RSVP) in infants, young children, and the elderly, accounting for most pediatric hospitalizations. In adults, RSV is also an underrecognized cause of deterioration in health^3^. Despite intensive research on the treatment and control of RSV, vaccines and specific therapies remain unavailable.

Natural products are a major source of antiviral drug discovery^4,5^. Quercetin (QR) is a common bioflavonoid found in many vegetables, fruits, and Chinese herbs. QR has been reported to possess numerous beneficial medicinal properties, such as its antiproliferative, antioxidant, antibacterial, and anticancer effects^6–9^. In addition, QR has been reported to exert inhibitory effects on the influenza, dengue, Ebola, Japanese encephalitis, hepatitis B, and hepatitis C viruses. The mechanisms of these inhibitory effects include activation of the transcription of interferon (IFN)-stimulated genes, reduction of oxidative stress, and the repression of HCV-induced upregulation of diacylglycerol acyltransferase (DGAT)^10–15^. Several studies have shown that QR also has an inhibitory effect on the RSV virus, although the mechanism of action is unclear^16,17^. Therefore, we aimed to conduct systematic and in-depth studies on the role of QR in RSV-induced lung inflammatory injury and investigate its underlying mechanism.

As a new area of pharmacology, network pharmacology provides new methods for elucidating the multiple mechanisms of actions of drugs by exploring the disease targets^18^. Metabolomics is also an emerging method in the field of systems biology, which can be used to analyze metabolites in biological tissues, cells, and fluids, provide global metabolic profiles, and identify differential metabolites^19,20^. In recent years, the combination of metabolomics and network pharmacology studies has been recognized as one of the most promising methods for assessing the mechanism of action of various therapeutic molecules^21–23^.

In this work, the effect of QR on RSV-induced lung inflammatory injury was confirmed using a mouse model. Subsequently, metabolites in the mouse lung tissues were identified by untargeted metabolomics to identify differential metabolites and metabolic pathways. Then, network pharmacology was performed to predict potential therapeutic targets of QR. Finally, the results of the metabolomics investigation and network pharmacology prediction were integrated to screen out the targets and metabolic pathways. The results of this research will provide useful insights into the mechanism of QR in RSVP treatment.

## Materials and methods

### Drugs and reagents

QR (C15H10O7, purity > 99.1%, #1000081) was obtained from National Institutes for Food and Drug Control (Beijing, China). Human RSV strain was obtained from the Institute of Viruses, Wuhan University (Wuhan, China). RSV antibody were purchased from ABCAM (U.K.). ELISA kits for mouse IL-1β and IL6 were bought from JIYI Biological Co., Ltd (Nanjing, China). Relevant primers were purchased from SANGON (Shanghai, China); other reagents were purchased from FUMAISI Biological Co., Ltd (Nanjing, China). Ribavirin, selected as positive drug control, was purchased from SICHUANBAILI Pharmaceutical Co., Ltd (Chengdu, China). Isoflurane were bought from RingPu Biological Co., Ltd (Tianjin, China). Ammonium acetate was purchased from Sigma Aldrich (Missouri, U.S.), Acetonitrile was purchased from Merck (New Jersey, U.S.), ammonium hydroxide and methanol were purchased from Fisher (Massachusetts, U.S.).

### Protocol of animal experiments in vivo

Forty specific pathogen-free (SPF) grade female BALB/c mice (age: 5–6 weeks) were purchased from Jiangsu Qinglongshan Co., Ltd. (Jiangsu, China). The mice were acclimated to a 12-h light–dark cycle for 7 days and given plenty of clean pellet feed and water. Next, the animals were randomly divided into 5 groups with 8 mice in each group: the control group (Con), the RSV-induced Model group (Mod), QR low-dose (QR-LD) group (50 mg/kg/d), QR high-dose (QR-HD) group (100 mg/kg/d), and the ribavirin control group (Riba) (46 mg/kg/d)^24,25^. All mice except those in the control group were infected with the RSV virus [1.4 × 107 plaque-forming units (PFU)] by intranasal instillation under mild anesthesia (isoflurane, 2%). Mice in the control group were administered an equal volume of 0.9% sodium chloride solution. Forty-eight hours after infection, drugs were administered orally to the treatment groups. The control and model groups were given an equal volume of 0.9% sodium chloride solution. Dosing was continued for 3 days, and the health status of the mice monitored by daily observation. On day 5 following infection, mice were sacrificed to collect relevant samples^26^. All animal experiments were approved by the Institutional Animal Care and Use Committee of Laboratory Animal Services Center at Nanjing University of Chinese Medicine (approval ID: SYXK (Su) 2018–0049) and performed according to the relevant guidelines and regulations, also all methods are reported in accordance with ARRIVE guidelines.

### Pathological and immunohistochemical examination

Mouse lung tissue was fixed with 4% paraformaldehyde solution and dehydrated after 24 h. The samples of mice were paraffin-embedded and cut into 3- mm thick sections. The sections were stained with hematoxylin and eosin (HE) and tested under a microscope in a double-blinded manner. Pathological changes include lung consolidation, thickening of the alveolar wall and lymphocyte infiltration. The pathological change score was expressed as 0–3 points, and the higher the score, the more serious the pathological change^27^.

### Immunofluorescence assay

Immunofuorescence was carried out using lung tissues. Lung sections were prepared for immunofuorescence after deparafnized, dehydrated and antigen retrieval. Next, the fixed tissue samples were mounted on the cover glass, blocked with donkey serum (Solarbio, Beijing, China) and probed with RSV-antibody. After rinsing thrice with PBS, the fixed tissue sections were incubated with the secondary antibody at 37 °C for 50 min in the dark. Afterwards, the cell nuclei were stained with 4′,6-diamidino-2-phenylindole at 37˚C for 10 min in the dark. Eventually, the sectioned tissues were covered with an anti-fade mounting medium for fuorescence microscopy (Olympus).

### Enzyme-linked immunosorbent assay (ELISA)

The concentrations of interleukin (IL)-1β and IL6 in lung tissues and serum were measured using ELISA kits according to the manufacturer’s protocol. Cytokine levels were determined based on the absorbance at 450 nm measured using a microplate reader.

### Quantitative real-time PCR

Total RNA was extracted from the lung tissue using the Trizol reagent (Thermo Fisher Scientific, USA), and extracted RNA was reverse-transcribed into cDNA using a reverse transcription and cDNA synthesis kit (ABM, Canada), according to the manufacturer’s instructions. Quantitative real-time PCR (QPCR) was conducted under QuantStudio 7 Flex Real-Time PCR System (Thermo Fisher Scientific, USA). The relative expression of mRNA was calculated by the 2 − ΔΔCT method. The specific primer sequences were used in Supplementary Table S1.

### Metabolomics study

#### Sample preparation

Lung tissues from Con, Mod, and QR (QR-HD) groups were used for metabolomics analysis. These tissues were quickly frozen in liquid nitrogen immediately after dissection, cut on dry ice (~ 80 mg), and transferred to Eppendorf tubes (2 mL). Each tissue sample was taken with 200 μL of H_2_O and five ceramic beads for homogenization, following which 800 μL of methanol/acetonitrile (1:1, v/v) were added to the homogenate for metabolite extraction. The mixture was centrifuged for 20 min (14,000*g*, 4 °C), and the supernatant was dried in a vacuum centrifuge. For LC–MS analysis, the dried samples were redissolved in 100 μL of an acetonitrile/water (1:1, v/v) mixture, centrifuged at 14,000*g* at 4 °C for 15 min, and then the supernatant was injected.

#### Ultra-high performance liquid chromatography (UHPLC)-Q-exactive orbitrap MS

Metabolomics analysis was performed using a UHPLC system coupled to a Q Exactive hybrid quadrupole Orbitrap mass spectrometer (Thermo Fisher Scientific, CA, United States). For hydrophilic interaction liquid chromatographic separation, samples were analyzed using a 2.1 mm × 100 mm ACQUIY UPLC BEH Amide 1.7 um column (waters, Ireland). In both ESI positive and negative modes, the mobile phase consisted of A = 25 mM ammonium acetate and 25 mM ammonium hydroxide in water and B = acetonitrile. The gradient consisted of 98% B for 1.5 min; it was linearly reduced to 2% in 10.5 min, maintained for 2 min, and then increased to 98% in 0.1 min, with a 3 min re-equilibration period. The electrospray ionization (ESI) source conditions were set as follows: Ion Source Gas 1 (Gas 1) as 60, Ion Source Gas 2 (Gas 2) as 60, curtain gas (CUR) as 30, source temperature: 600 °C, Ion Spray Voltage Floating ± 5500 V.

##### Data processing and analysis

The raw MS data were converted to MzXML files using ProteoWizard MSConvert before importing into freely available XCMS software. For peak picking, the following parameters were used: centWave m/z = 10 ppm, peak width = c (10, 60), and prefilter = c (10, 100). For peak grouping, bw = 5, mzwid = 0.025, and minfrac = 0.5 were used. The Collection of Algorithms of MEtabolite pRofile Annotation (CAMERA) was used for the annotation of isotopes and adducts. In the extracted ion features, only the variables having more than 50% of the nonzero measurement values in at least one group were kept. Compound identification of metabolites was performed by comparing the accuracy of the *m/z* values (< 10 ppm), and MS/MS spectra were compiled into an in-house database established with available authentic standards.

After sum-normalization, the processed data were analyzed using SIMCA 14.0 software (Umetrics, Umea, Sweden), where the data were subjected to multivariate data analysis, including Pareto-scaled principal component analysis (PCA) and partial least squares discriminant analysis (PLS-DA). The significantly differential metabolites were identified based on the threshold of fold change (FC) > 1.2 (or < 0.83) and *p* < 0.05. Metabolite enrichment analysis and pathway analysis were performed using Metaboanalyst 5.0 (https://www.metaboanalyst.ca)^28^.

### Network pharmacology analysis

#### Data preparation and potential therapeutic target analysis

Target proteins of QR were obtained in the TCMSP database (https://old.tcmsp-e.com/tcmsp.php)^29^. The SMILES structural formula of QR was downloaded from PubChem database (https://www.ncbi.nlm.nih.gov/pccompound)^30^. The SMILES structural formula was input in the SwissTargetPrediction database (http://swisstargetprediction.ch/)^31^, PharmMapper database (http://lilab-ecust.cn/pharmmapper/)^32^ to obtain the target proteins, and SEA database (http://targetnet.scbdd.com/home/index/) to obtain the corresponding target genes^33^. The corresponding target genes were obtained associated with their target proteins through the String database (https://string-db.org/)^34^ and UniProt databases (https://www.uniprot.org/)^35^.

We used GeneCards (https:www.genecards.org/), DisGeNET database (http://www.disgenet.org/) and Online Mendelian Inheritance in Man (OMIM, https://www.omim.org/) databases to search disease targets of RSVP^36–38^. The search term was “respiratory syncytial virus pneumonia,” “RSV pneumonia.” and “RSV-induced lung inflammation.” Retrieval results were combined after deleting duplicates to obtain the RSVP targets. R software was used to analyze the interaction between QR targets and RSVP targets to obtain potential therapeutic targets. The results are presented in a Venn diagram^39^. Cytoscape 3.6.0 software was used to construct a drug-target-disease network diagram to comprehensively analyze the molecular mechanism of QR in the treatment of RSVP^40^.

#### Protein–protein interaction network analysis and biological function analysis

The STRING database (https://string-db.org/) aims to collect and integrate all publicly available sources of protein–protein interaction (PPI) information and achieve a comprehensive and objective network^41^. We uploaded the potential therapeutic targets and drew a PPI network graph by the Cytoscape software.

To illustrate the role of potential therapeutic targets in gene function and signaling pathways, we used the R package clusterProfiler for Gene Ontology (GO) function enrichment analysis and Kyoto Encyclopedia of Genes and Genomes (KEGG) pathway enrichment analysis^42,43^. The GO function enrichment analysis, including biological process (BP), cellular component (CC), and molecular function (MF) items, was conducted, and we plotted the top ten relevant GO enrichment items as bubble plots. The KEGG is a reference knowledge base for the biological interpretation of genome sequences, and we plotted the top twenty relevant KEGG pathways as bubble plots.

### Integrated analysis of metabolomics and network pharmacology

The identified differential metabolites were used to construct a compound-reaction-enzyme-gene (CREG) network using the MetScape plugin in Cytoscape 3.6.0 software. The potential therapeutic targets identified by network pharmacology were overlapped with those identified in the CREG network to obtain common targets for QR treatment of RSVP.

### Molecular docking simulation

The binding of the key targets to QR was evaluated by molecular docking. Molecular docking simulations were performed using AutoDock 4.2 and AutoDock Vina software according to published methods. The macromolecular protein target receptors were obtained from the PDB database, and 2D structures of small-molecule constituents were obtained from the PubChem Database (https://pubchem.ncbi.nlm.nih.gov/)^44^.

### Statistical analysis

GraphPad Prism 8.0 software was used for statistical analysis. All data obtained from at least three independent experiments were presented as the mean ± standard error. A one-way analysis of variance test was used to compare the means of different groups. Significance was set at 0.05 and 0.01.

## Results

### QR attenuated weight loss in RSV-infected mice

**Figure 1:**
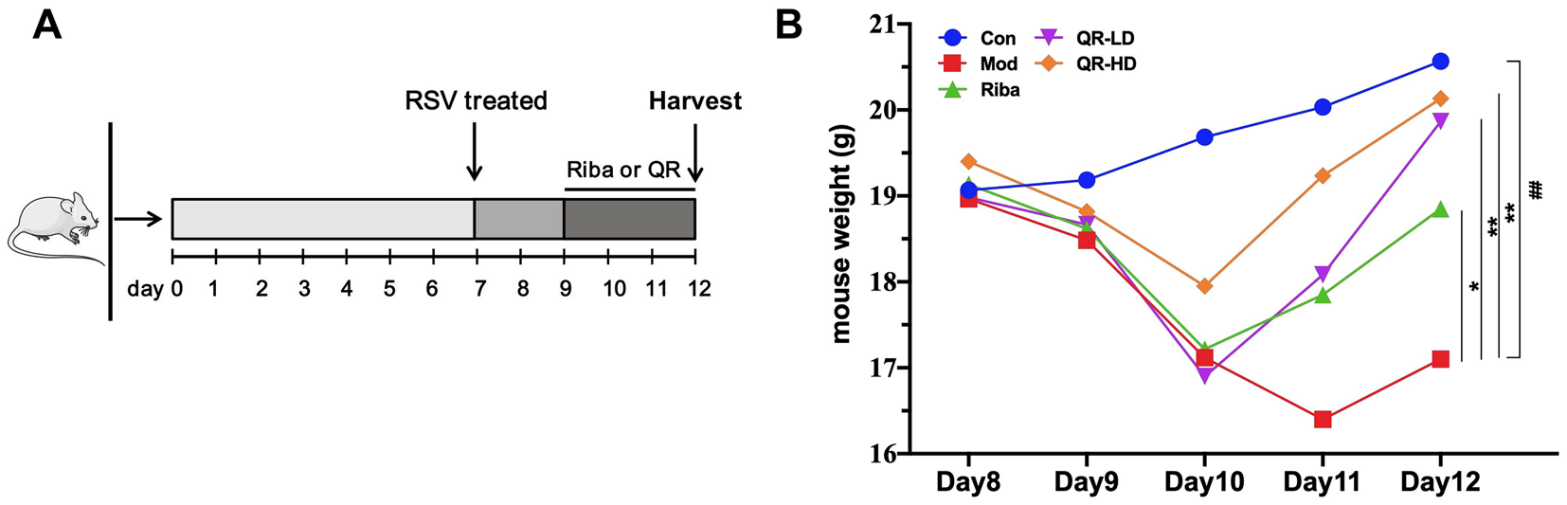
QR attenuated the weight loss in RSV-infected mice. **(A)** Experimental protocol. **(B)** Body weight changes of the mice. Significance: ## *P* < 0.01 vs control group; # *P* < 0.05 vs control group; ** *P* < 0.01 vs model group; * *P* < 0.05 vs model group.

Weight loss is a quantitative measure of the severity of RSV illness in the BALB/c mouse model. To assess the effects of QR on body weight loss in response to RSV infection, the mice were infected with a suspension containing RSV (Fig. 1A). After RSV infection, all the mice demonstrated a decrease in body weight, with marked weight loss occurring on day 10 and a gradual recovery of body weight by days 11–12. On day 12, compared with the control group, the mice in the model group had significantly lower body weight, and QR (50 mg/(kg∙d), 100 mg/(kg∙d)) was able to attenuate the weight loss of the infected mice (Fig. 1B).

### QR mitigated RSV-induced lung injury and inhibited virus replication

**Figure 2:**
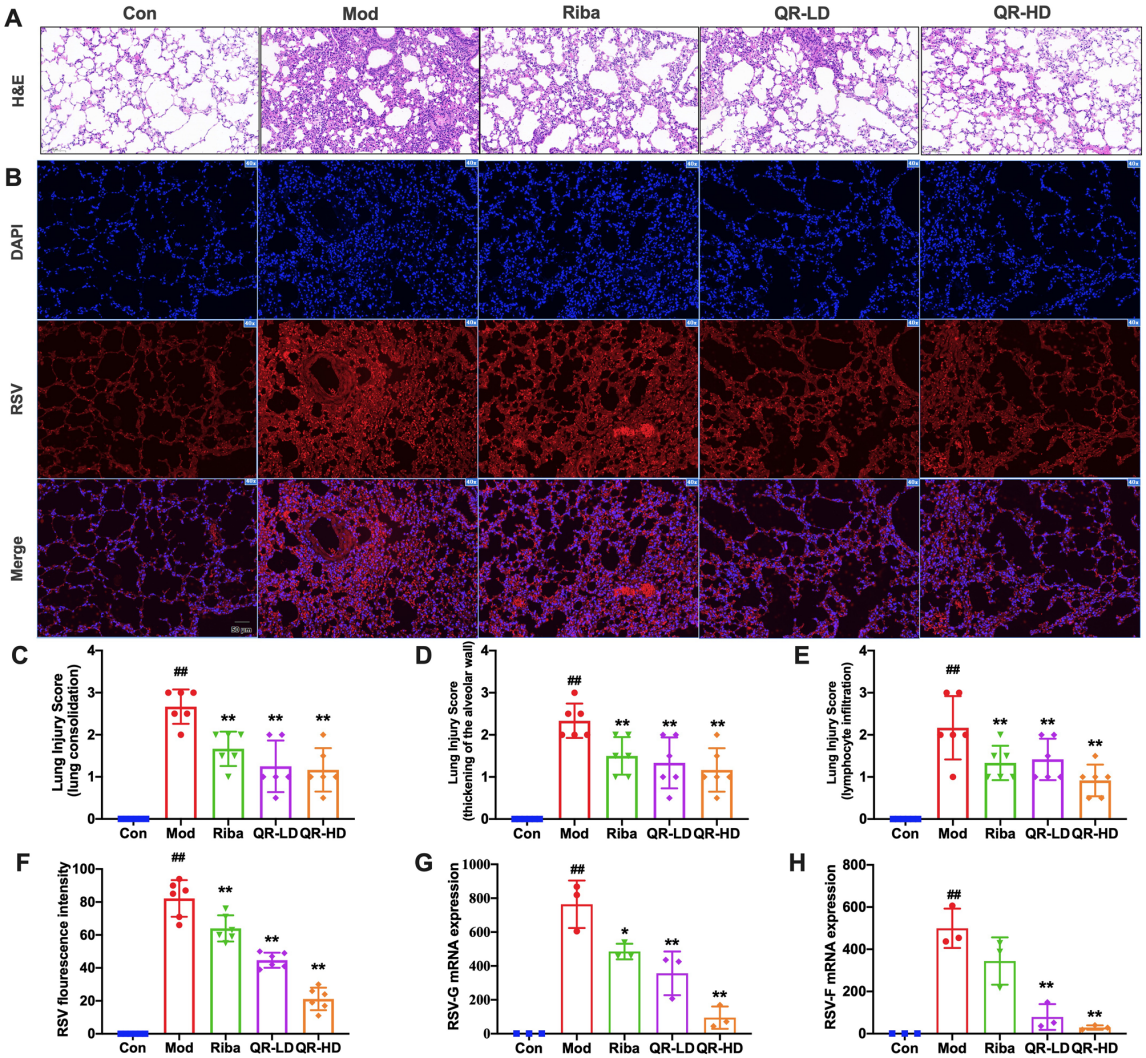
QR mitigated RSV-induced pulmonary histopathological damage and inhibited virus replication. **(A)** Pathological changes in lung tissue induced by RSV. Scale bar, 100 μm. **(B)** RSV expression assessed via an immunofluorescence assay. Scale bar, 50 μm. **(C–E)** Lung injury scores according to the degree of lung damage. **(F)** RSV fluorescence intensity. **(G,H)** RSV-G and RSV-F mRNA levels. Data are presented as the mean ± standard error. Significance: ## *P* < 0.01 vs control group; # *P* < 0.05 vs control group; ** *P* < 0.01 vs model group; * *P* < 0.05 vs model group.

The histological changes in the lung tissue were investigated by H&E staining. As indicated in Fig. 2A, the lung tissue of mice from the control group exhibited clear alveolar lobules and alveolar cavities without leakage or cell infiltration in the alveolar spaces or the interstitium. However, RSV infection caused severe pulmonary inflammation characterized by lung consolidation, thickening of the alveolar wall, and lymphocytic infiltration. RSV infection also caused inflammatory cell infiltration into the lung interstitium and alveolar space. Compared with the model group, the pathological damage and lung injury scores (lung consolidation, thickening of the alveolar wall, and lymphocyte infiltration) in the QR-treated groups were notably reduced, with mild inflammatory cell infiltration and protein leakage in the alveolar cavity (Fig. 2C–E).

The severity of RSV infection is associated with the level of virus amplification. To confirm RSV virus replication in the lung, the relative expression of RSV-G and RSV-F genes was detected by QPCR. As shown in Fig. 2G,H, RSV infection resulted in a marked increase in RSV-G and RSV-F mRNA levels in mouse lung tissue. In the QR-treated groups, the mRNA levels of RSV-G and RSV-F were significantly decreased in a dose-dependent manner compared to those of the model group. To monitor the challenge dose of the virus in mouse lung, we also determined the virus levels in the lung by immunofluorescence assay (Fig. 2B). The results of the quantitative analysis showed that virus levels in the QR-treated groups were drastically reduced compared with that in the model group (Fig. 2F). Overall, these results indicated that QR effectively alleviated lung injury and inhibited virus replication in RSV-infected mice.

### QR reduced pro-inflammatory cytokine expression in RSV-infected mice

**Figure 3:**
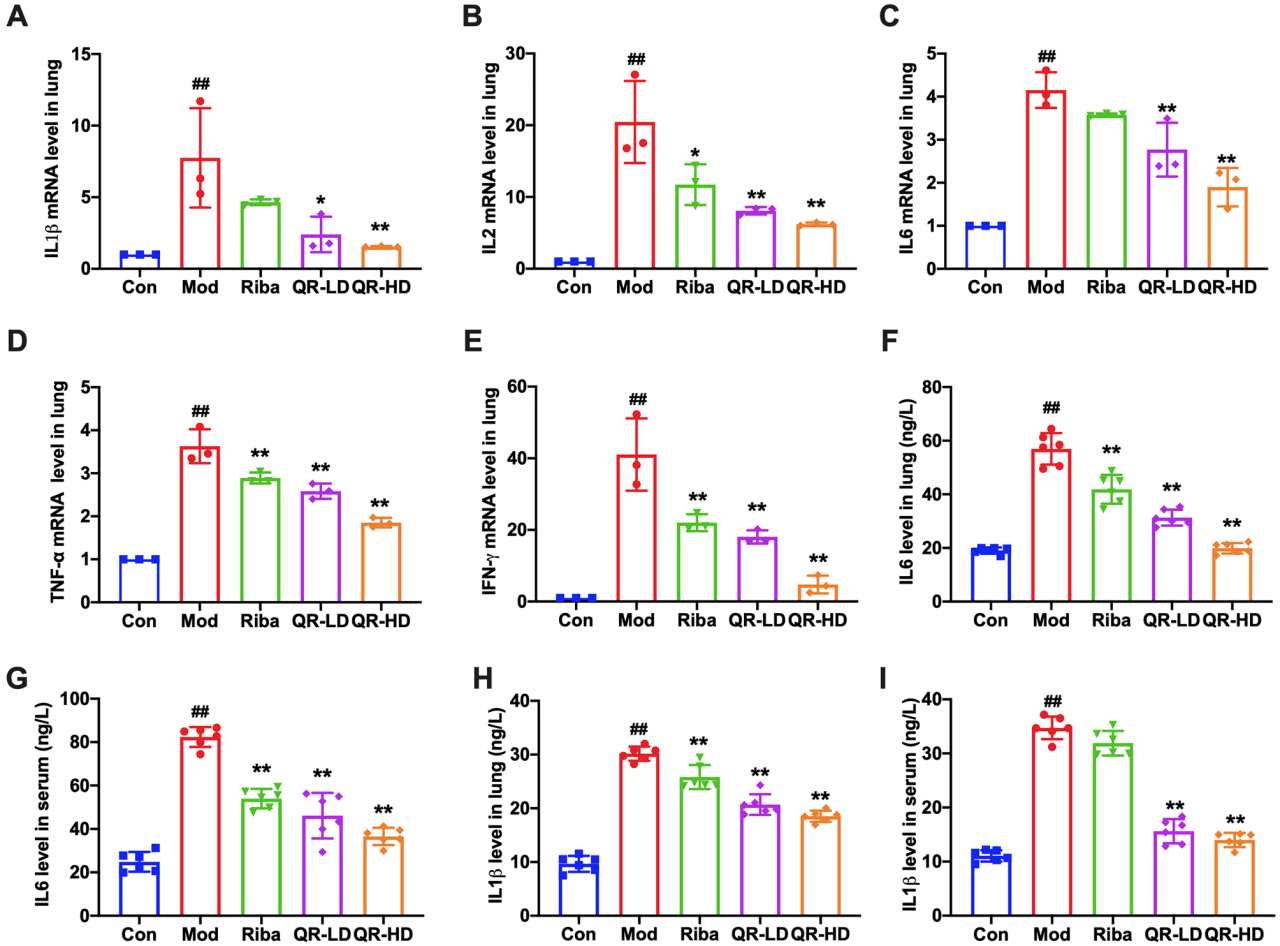
QR reduced the production of pro-inflammatory cytokines in lung tissues of RSV-challenged mice. **(A–E)** mRNA Levels of IL1β, IL2, IL6, TNF-α, and IFN-γ in lung tissue. **(F–I)** Levels of IL1β and IL6 in lung tissue and serum. Data are presented as the mean ± standard error. Significance: ## *P* < 0.01 vs control group; # *P* < 0.05 vs control group; ** *P* < 0.01 vs model group; * *P* < 0.05 vs model group.

RSV induces the expression of pro-inflammatory cytokines, including IL1β, IL2, IL6, TNF-α, and IFN-γ, which contribute to inflammation and the pathology of the infection^45^. Therefore, we evaluated the mRNA levels of these inflammatory cytokines, IL1β, IL2, IL6, TNF-α, and IFN-γ, in lung tissues. The results in Fig. 3A–E show that the mRNA levels of IL1β, IL2, IL6, TNF-α, and IFN-γ in the lung tissues of the model group were significantly increased compared to those of the control group. In the QR-treated groups, the mRNA levels of these genes were significantly decreased in a dose-dependent manner compared to those of the model group. To further explore the inhibitory effect of QR on lung inflammation in the mice, we also measured the effects of QR on IL-1β and IL6 secretion in mouse lung homogenate and serum. The results showed that the protein levels of IL-1β and IL6 of the model group were significantly increased compared to those of the control group. In the QR-treated groups, at doses of 50 mg/(kg∙d) and 100 mg/(kg∙d), levels of these inflammatory factors were significantly decreased in a dose-dependent manner compared to those of the model group (Fig. 3F–I).

### Metabolic profiles of lung tissue samples

**Figure 4:**
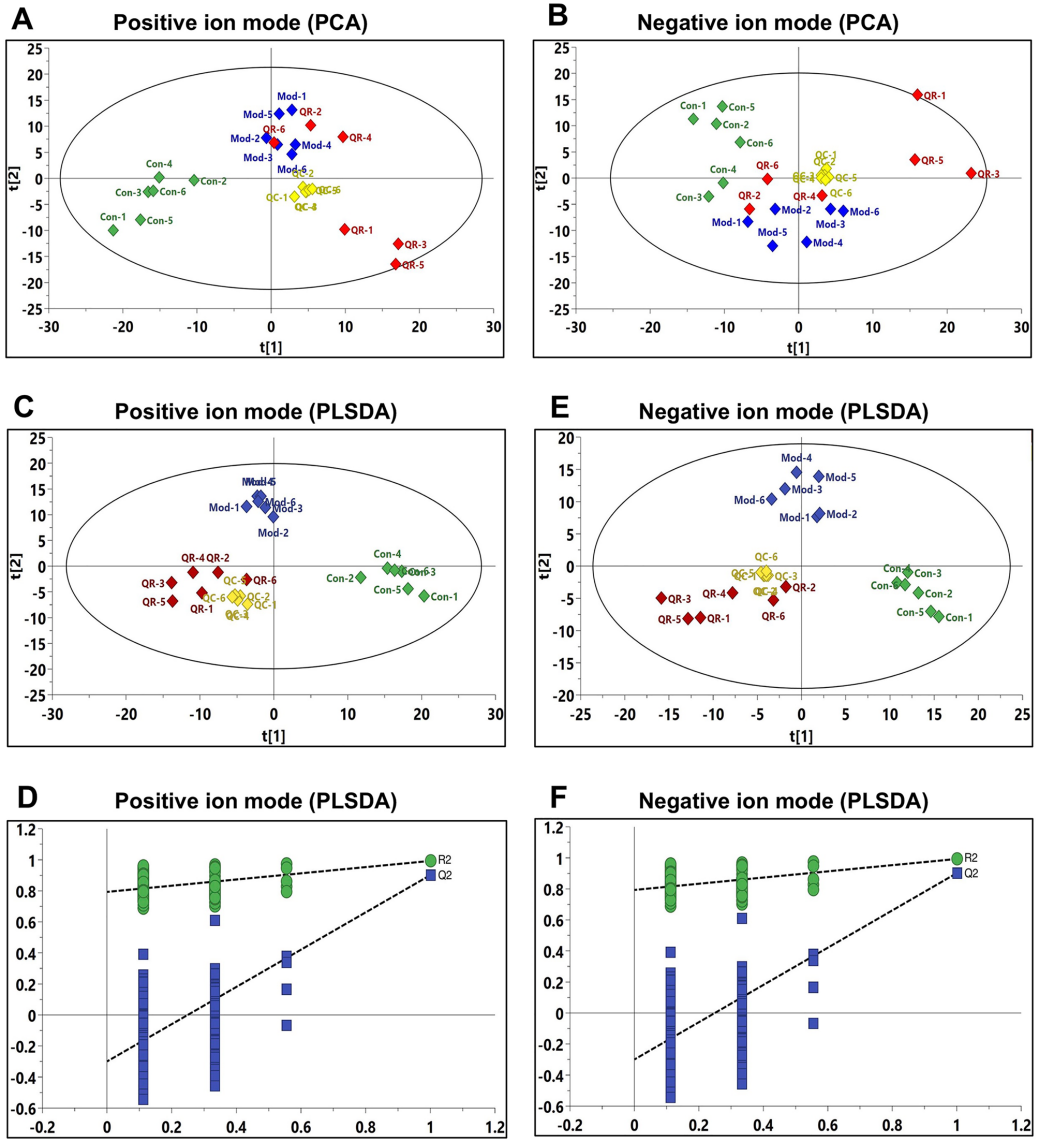
Multivariate data analysis of lung tissue metabolites. **(A,B)** PCA score plot in the positive and negative ion modes (n = 6). **(C,D)** PLS-DA score plot in the positive and negative ion modes (n = 6). **(E,F)** PLS-DA permutation test graph in the positive and negative ion modes (n = 200).

The data of each group were assessed using unsupervised principal component analysis (PCA), which showed the Con and Mod groups to be significantly separated (Fig. 4A,B). The trend indicated differences in metabolic profiles between groups—significant changes in lung tissue endogenous metabolites—caused by RSV infection. The QC samples showed good aggregation, and the results indicated that the entire analysis system had good stability and repeatability, meeting the requirements for metabolomics analysis. Then, a partial least squares discriminate analysis (PLS-DA) model was applied to identify the clustering property. As shown in Fig. 4C–E, PLS-DA score plots showed relatively tight clusters and clear discrimination among Con, Mod, and QR groups in both positive and negative ion modes. This indicated that RSV infection and QR treatment caused significant changes in metabolites. Subsequently, permutation tests were utilized to validate the established PLS-DA model. As shown in Fig. 4D–F, the model had good R^2^ and Q^2^ values after 200 response permutation tests, indicating that the PLS-DA model was reliable with a low risk of overfitting.

### Identification of differential metabolites

**Figure 5:**
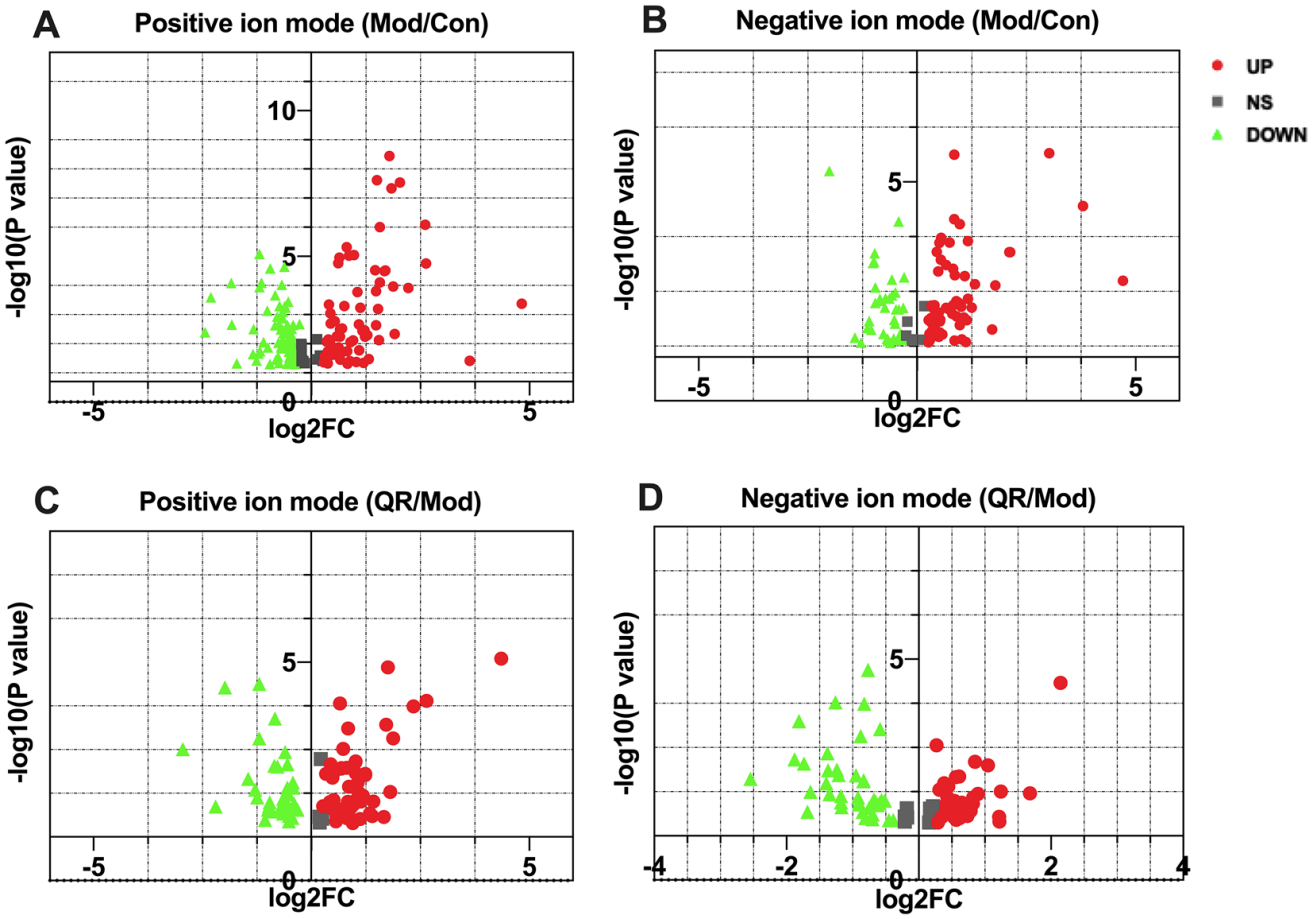
Volcanic map of the identified differential metabolites. **(A,B)** In Mod and Con groups, 239 metabolites were identified. **(C,D)** In QR and Mod groups, 160 metabolites were identified. P values were all < 0.05.

With a threshold of FC > 1.2 or < 0.83 and p < 0.05, a total of 239 metabolites were identified between the Mod and Con groups, including 143 metabolites in the positive ion mode and 96 metabolites in the negative ion mode (Fig. 5A,B). A total of 160 metabolites were identified between the QR and Mod groups, including 88 metabolites in the positive ion mode and 72 metabolites in the negative ion mode (Fig. 5C,D). A total of 52 intersectant metabolites (28 metabolites in the positive ion mode and 24 metabolites in the negative ion mode) were obtained by overlapping the differential metabolites between Mod versus Con and QR versus Mod groups. As shown in Table 1, compared with the Con group, the levels of 25 metabolites increased and the levels of 27 metabolites decreased in the model group. After QR therapy, the levels of these metabolites were restored to normal levels to various extents.

**Table 1:** Different endogenous metabolites.

No	Metabolites	Retention time (s)	Mod/Con	QR/Mod	Scan model
1	1-hexadecyl-2-(8z,11z,14z-eicosatrienoyl)-sn-glycero-3-phosphocholine	166.493	↑##	↓*	+
2	2'-deoxycytidine	229.9805	↑##	↓**	+
3	Beta-alanine	51.4713	↑#	↓**	+
4	Erythromycylamine	48.3226	↑#	↓*	+
5	Hydrocortisone	195.005	↑##	↓*	+
6	N-acetyl-.beta.-d-mannosamine	279.965	↑#	↓*	+
7	Procymidone	247.893	↑##	↓*	+
8	Stachydrine	290.147	↑#	↑*	+
9	1-Oleoyl-sn-glycero-3-phosphocholine	211.7445	↓##	↑**	+
10	4-fluoroisocathinone	107.0669	↓##	↑**	+
11	4-nitroaniline	167.349	↓##	↑**	+
12	Aflatoxin g1	185.814	↓##	↑**	+
13	Batimastat	105.916	↓##	↑**	+
14	Fenfluramine	186.373	↓##	↑**	+
15	Fingolimod	62.73815	↓#	↓*	+
16	Glycerophosphocholine	404.382	↓##	↑**	+
17	Homatropine	291.7625	↓#	↓*	+
18	Hypoxanthine	186.545	↓##	↑**	+
19	Methanone, [1-(6-fluorohexyl)-1 h-indol-3-yl]-1-naphthalenyl-	298.2525	↓##	↓**	+
20	Methyl (1-(cyclohexylmethyl)-1 h-indole-3-carbonyl)-l-valinate	81.73895	↓##	↑**	+
21	N-.alpha.-acetyl-l-ornithine	186.464	↓##	↑*	+
22	Napelline	237.909	↓#	↓*	+
23	O-desmethylmycophenolic acid	260.315	↓#	↑**	+
24	Oxybutynin	238.584	↓#	↓*	+
25	Prometryne	185.183	↓##	↑*	+
26	S-adenosyl-l-methionine	468.195	↓#	↑*	+
27	S-methyl-5'-thioadenosine	95.5725	↓##	↑**	+
28	Zaleplon	225.9345	↓##	↑**	+
29	(1-acetyloxy-3-hydroxy-6,8a-dimethyl-7-oxo-3-propan-2-yl-2,3a,4,8-tetrahydro-1 h-azulen-4-yl) 4-hydroxybenzoate	34.7114	↑#	↓*	−
30	2-chloro-l-phenylalanine	188.61	↑##	↓**	−
31	4-nitrocatechol	163.0735	↑#	↓**	−
32	4'-hydroxydiclofenac	400.1335	↑#	↓**	−
33	5a,6-anhydrotetracycline	265.865	↑#	↓*	−
34	Borrelidin	215.7105	↑##	↑**	−
35	D-gluconate	98.9872	↑##	↓*	−
36	Deoxycytidine	231.029	↑##	↓**	−
37	Embelin	23.721	↑##	↓*	−
38	Fenhexamid	188.587	↑#	↓**	−
39	Hboa + o-hex	193.0975	↑#	↑**	−
40	His-ser	94.9091	↑##	↓**	−
41	Humulone	196.723	↑##	↓*	−
42	Ketoleucine	58.6159	↑##	↓*	−
43	UDP-N-acetylglucosamine	530.223	↑##	↑*	−
44	Uric acid	345.205	↑##	↓**	−
45	Zoledronic acid	345.6995	↑##	↓**	−
46	2'-hydroxy-3,4,6'-trimethoxychalcone	239.2355	↓#	↑*	−
47	Adenosine 5'-monophosphate	521.803	↓#	↑*	−
48	Dehydroascorbic acid	97.6174	↓##	↑**	−
49	Melatonin	46.2645	↓##	↓*	−
50	Nilutamide	466.985	↓##	↑*	−
51	Testosterone	101.698	↓#	↑*	−
52	Topiramate	350.416	↓##	↑*	−

↑ represents increase, ↓ represents reduction. ## *P* < 0.01 and # *P* < 0.05, Mod group versus Con group. ** *P* < 0.01 and * *P* < 0.05, QR group versus Mod group.

### Analysis of metabolic pathways

**Figure 6:**
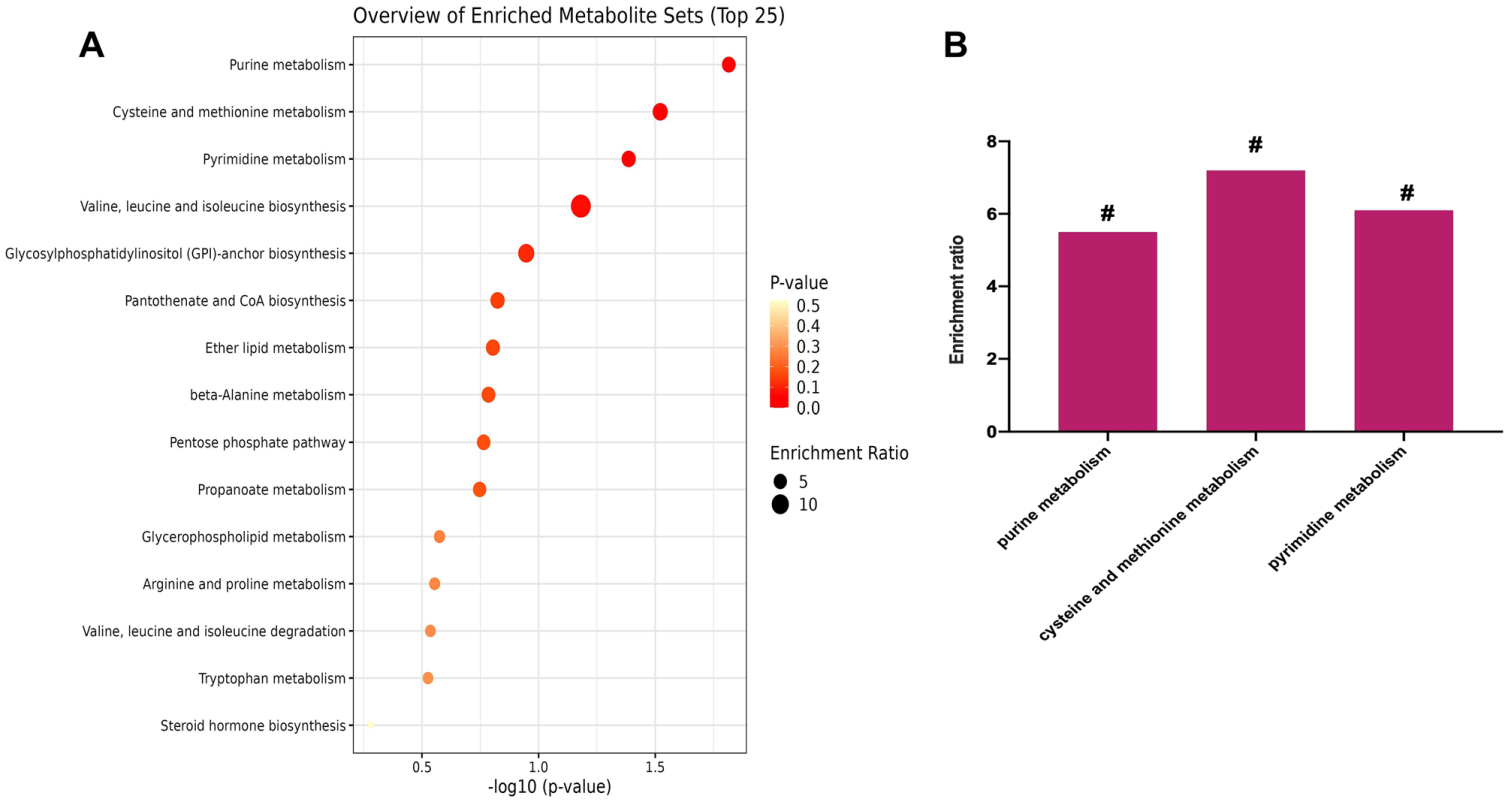
Analysis of Metabolic Pathways. **(A)** Metabolic pathway enrichment analysis of differential metabolites. Node size is based on enrichment ratio; node color is based on P value. **(B)** Enrichment ratios of three important metabolic pathways.

The 52 metabolites were imported into the Metaboanalyst 5.0. for metabolic approach analysis, and P < 0.05 was used as the screening basis. As a result, a total of three important metabolic pathways, including purine metabolism, cysteine and methionine metabolism, and pyrimidine metabolism were screened (Fig. 6A,B). The relevant metabolites involved were adenosine 5'-monophosphate, hypoxanthine, uric acid, *S*-adenosyl-l-methionine, *S*-methyl-5'-thioadenosine, deoxycytidine, and beta-alanine.

### Potential therapeutic targets of QR in the treatment of RSVP

Based on QR chemical structure, the TCMSP, SwissTargetPrediction, PharmMapper, and SEA databases were used to predict the potential target genes of QR. A total of 317 target genes were collected from the TCMSP (n = 151), SwissTargetPrediction (n = 100), PharmMapper (n = 296) and SEA (n = 150) databases, and a total of 518 targets were obtained after combination and deduplication.

To obtain RSVP targets, we searched the GeneCards, DisGeNET and OMIM databases. In total, 685 RSVP targets were retrieved from the GeneCards database, 2 RSVP targets from the DisGeNET database and 71 RSVP targets from the OMIM database. After combining the results of the three databases and removing the duplicates, a total of 740 RSVP targets were obtained.

**Figure 7:**
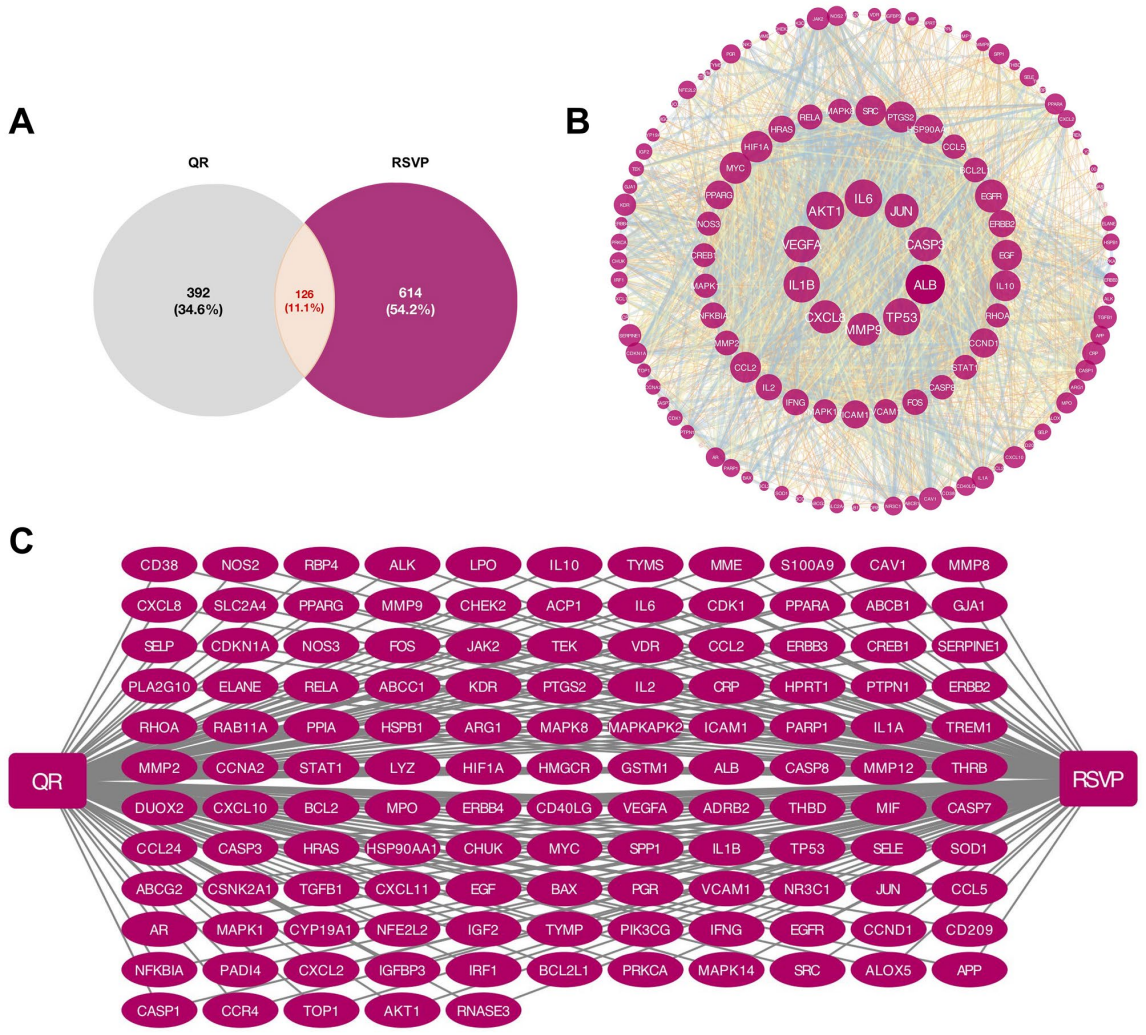
Prediction of the potential therapeutic targets and PPI Network Analysis. **(A)** Venn diagram of the potential therapeutic targets. **(B)** PPI analysis. **(C)** Drug-Target-Disease network diagram.

Next, potential therapeutic targets were obtained by overlapping the 518 QR targets and 740 RSVP targets. Ultimately, 126 potential therapeutic targets were obtained through this interaction analysis (Fig. 7A, Supplementary Table S2). At the same time, an interactive QR-target genes-RSVP network was constructed (Fig. 7C), which indicated that QR might have an effect on RSVP by stimulating or inhibiting these target genes.

### Protein–protein interaction network analysis and biological function analysis

One hundred and twenty-six potential therapeutic targets were imported into the STRING database for PPI network analysis and the PPI interaction network was constructed using the Cytoscape software. As shown in Fig. 7B, ALB, AKT1, IL6, TP53, VEGFA, IL1β, CASP3, JUN, CXCL8, and MMP9 were the top 10 genes with high degree values.

**Figure 8:**
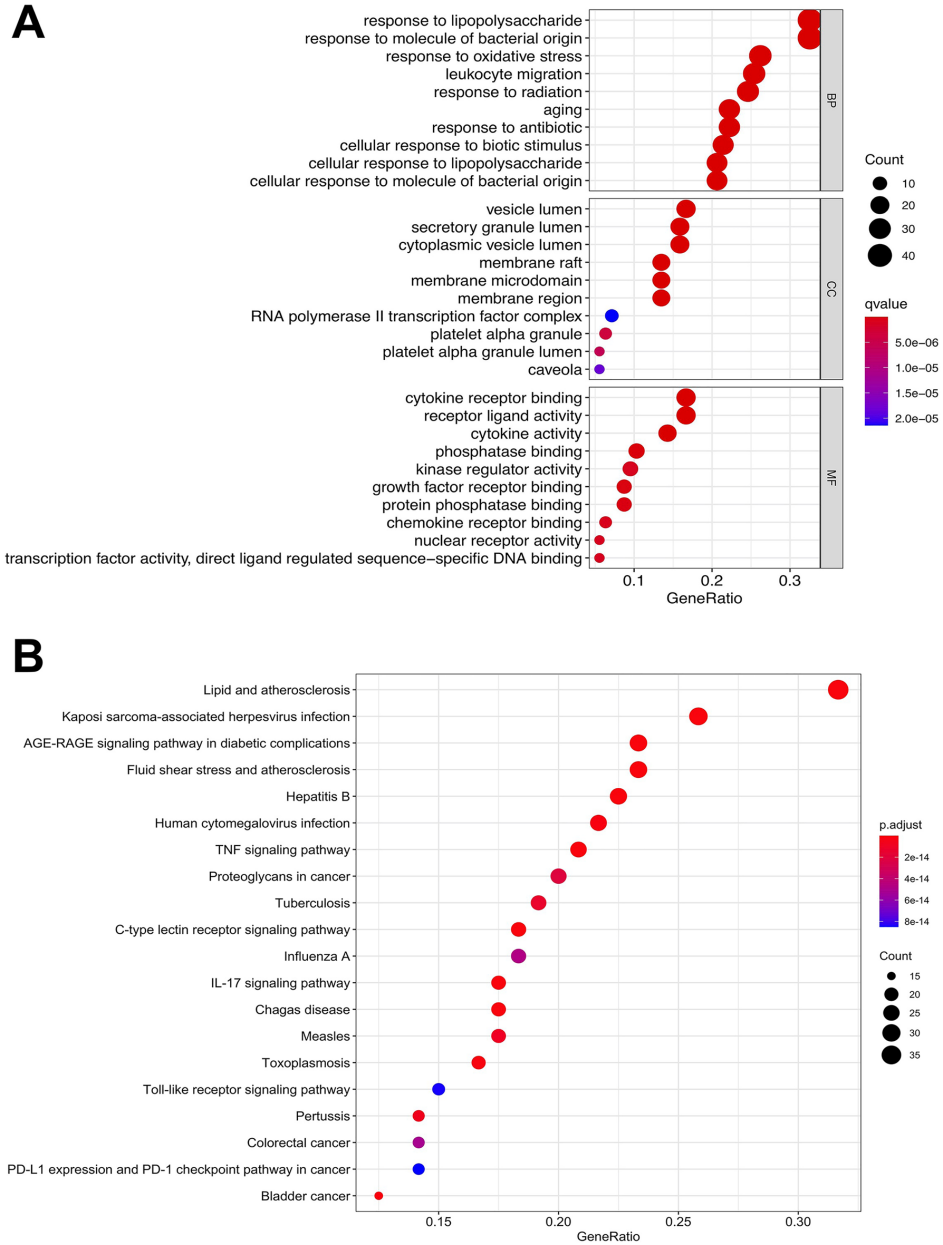
Biological function analysis. **(A)** GO and **(B)** KEGG enrichment analysis of the potential therapeutic targets.

Through GO function enrichment analysis, we obtained the top ten GO items of biological processes (BP), cellular components (CC), and molecular functions (MF). KEGG pathway enrichment analysis resulted in 20 KEGG pathways. The results of GO functional enrichment showed that the BP of potential therapeutic targets mainly included a response to lipopolysaccharides and molecules of bacterial origin. The CC of potential therapeutic targets mainly included vesicle lumen, secretory granule lumen, and cytoplasmic vesicle lumen. The MF of potential therapeutic targets mainly included cytokine receptor binding and receptor-ligand activity. The results of the KEGG enrichment analysis showed that the signaling pathways related to potential therapeutic targets mainly included the lipid and atherosclerosis signaling pathway, Kaposi's sarcoma-associated herpesvirus infection signaling pathway, and the AGE-RAGE signaling pathway (Fig. 8).

### Integrated analysis of metabolomics and network pharmacology

The identified, differential metabolites were imported into Metscape to obtain a compound-reaction-enzyme-gene (CREG) network, which displayed the interactions among metabolites, pathways, enzymes, and genes. As shown in Supplementary Table S3, 244 potential targets were found in the CREG network.

**Figure 9:**
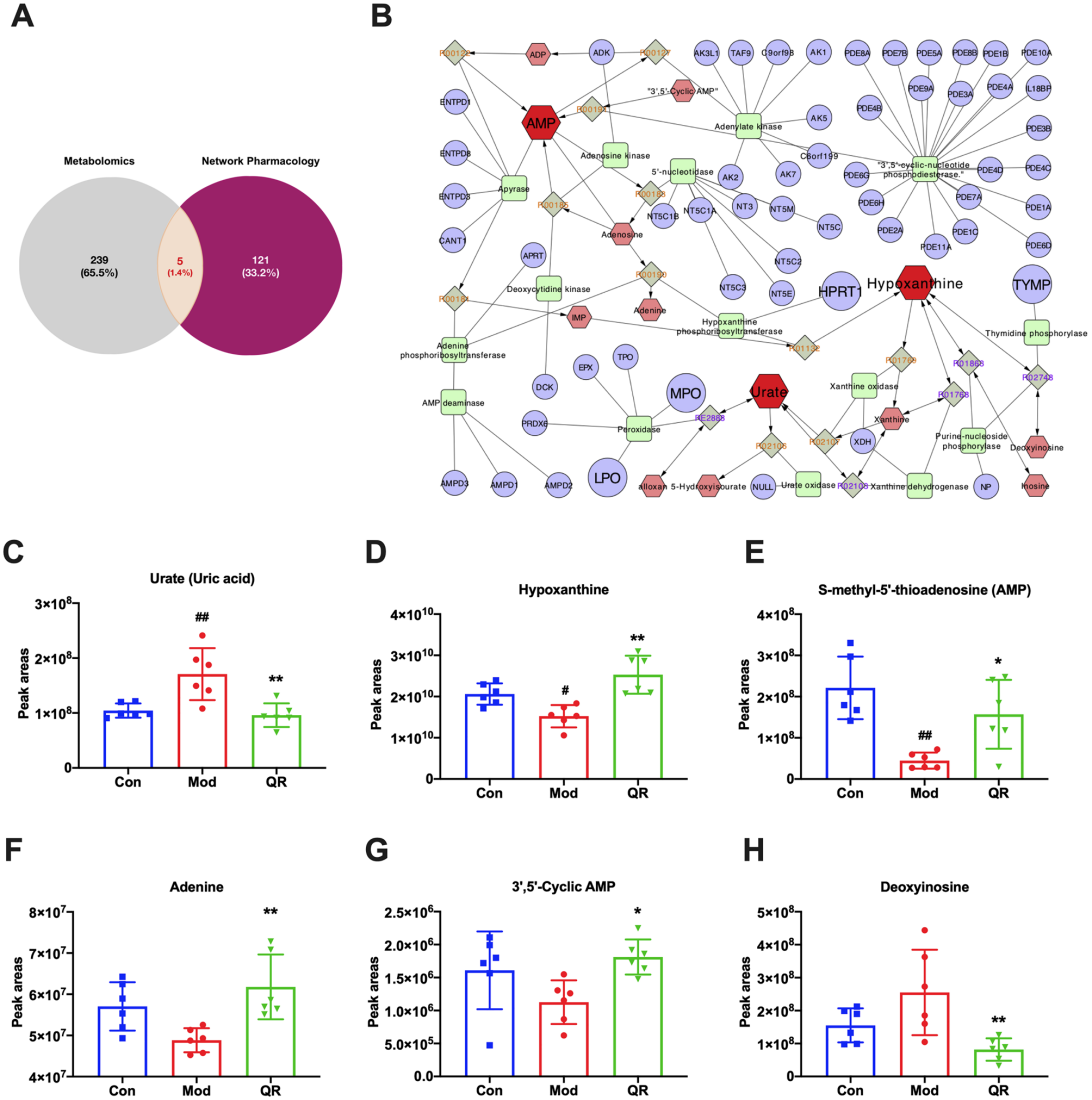
Integrated analysis of metabolomics and network pharmacology. **(A)** Venn diagram of the key targets. **(B)** The compound-reaction-enzyme-gene network of purine metabolism. The red hexagons, gray diamonds, green round rectangles, and purple circles represent active compounds, reactions, proteins, and genes, respectively. **(C–H)** The peak areas of metabolites. Data are presented as the mean ± standard error. Significance: ## *P* < 0.01 vs control group; # *P* < 0.05 vs control group; ** *P* < 0.01 vs model group; * *P* < 0.05 vs model group.

By intersecting 244 targets in the CREG network with 126 targets from the network pharmacology analysis (Fig. 9A), five common targets were identified, including hypoxanthine–guanine phosphoribosyltransferase (HPRT1), thymidine phosphorylase (TYMP), lactoperoxidase (LPO), myeloperoxidase(MPO), and cytochrome P450 19A1(CYP19A1).

As shown in Fig. 9B, HPRT1, TYMP, LPO, and MPO are components in the purine metabolism pathway. Combined with the results of the KEGG analysis of differential metabolites, purine metabolism was shown to be the key metabolic pathway modulated by QR in the treatment of RSVP, and adenosine 5'-monophosphate (AMP), hypoxanthine, and urate (uric acid) were the key, differential metabolites produced by the purine metabolism pathway.

Quantitative analysis of these key, differential metabolites showed that the levels of uric acid were significantly increased, whereas the levels of AMP and hypoxanthine were significantly decreased in the model group compared with the control group. Treatment with QR could reverse this trend. In addition, the levels of adenine and 3′5’-cyclic AMP were significantly increased, whereas the levels of deoxyinosine were significantly decreased in the QR-treated groups compared with the model group (Fig. 9C–H).

### Molecular docking and analysis

**Figure 10:**
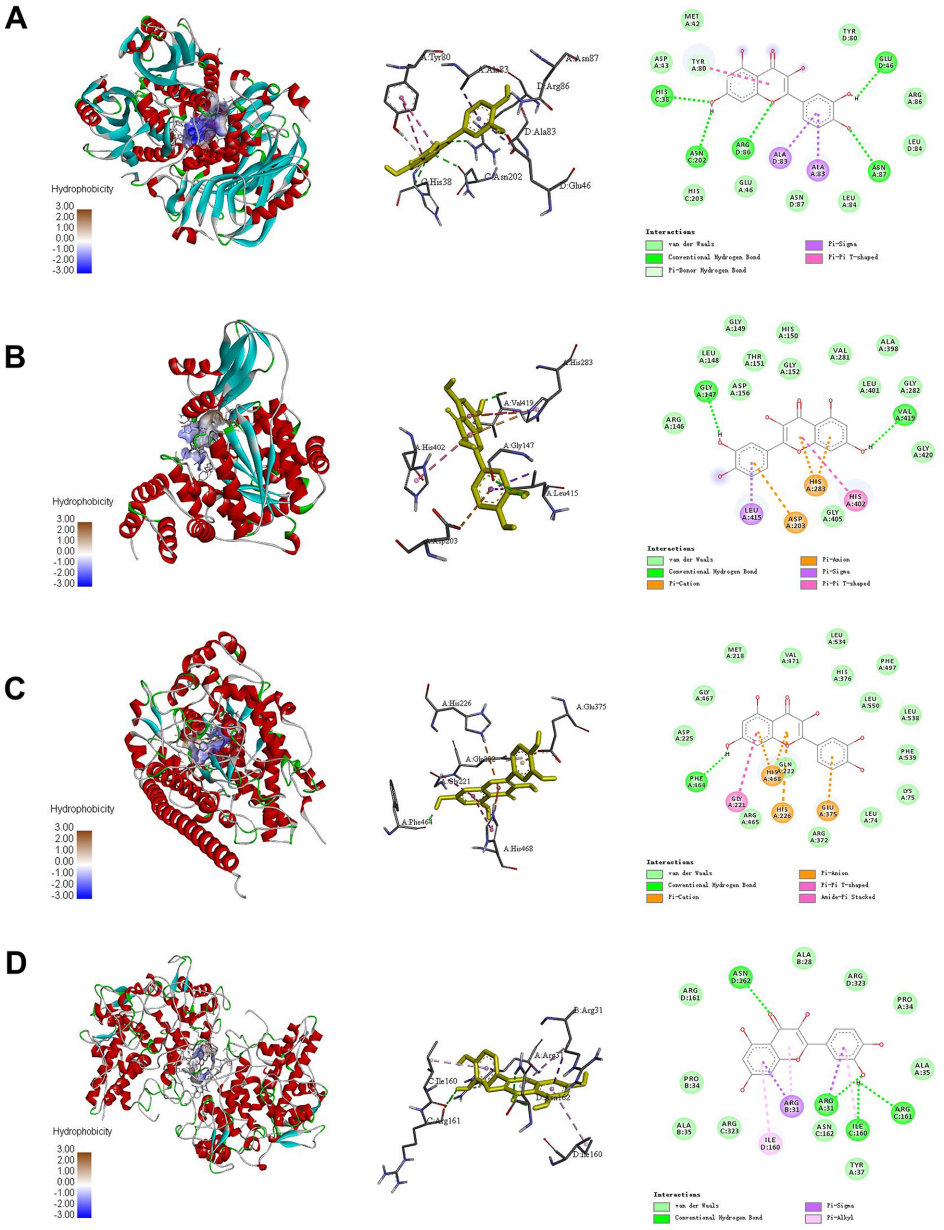
Molecular docking patterns of QR with key targets. **(A)** HPRT1-QR. **(B)** TYMP-QR. **(C)** LPO-QR. **(D)** MPO-QR**.** The crystal structure of key targets were obtained from RCSB Protein Data Bank (PDB, http://www.rcsb.org/). The PubChem (https://pubchem.ncbi.nlm.nih.gov/) was used to prepare the chemical structure of QR. The molecular docking was executed by AutoDock-Vina 1.1.2 (https://vina.scripps.edu/). The PyMOL 2.3.0 (https://pymol.org/2/) and BIOVIA Discovery Studio 2016 (http://www.discoverystudio.net/) were applied for results processing and visualization.

Molecular docking was used to mimic the binding ability of QR and key targets. Based on the above screening results, HPRT1, TYMP, LPO and MPO were verified. The 3D structure was imported into Autodock and docked with QR. The energy values of docking results were -7.7 kcal/mol, -7.7 kcal/mol, -8.9 kcal/mol, and -8.7 kcal/mol for binding of QR with HPRT1, TYMP, LPO, and MPO, respectively. These docking energy values were relatively small, indicating that QR could stably bind to the gene. QR mainly interacted with amino acid residues GLUD46, HISC38, ASNC202, ARGD86, ASNA87, and TYRA80 of HPRT1 (Fig. 10A). QR and TYMP were predicted to form a stable complex based on interactions of QR with the amino acid residues GLYA147, VALA419, and HISA402 (Fig. 10B). QR and LPO were predicted to form a stable complex based on interactions of QR with the amino acid residues PHEA464 and GLYA221 (Fig. 10C). In addition, QR mainly interacted with amino acid residues ASND162, ARGA31, ARGC161, and ILEC600 of MPO (Fig. 10D).

### Effects of QR on the key targets

**Figure 11:**
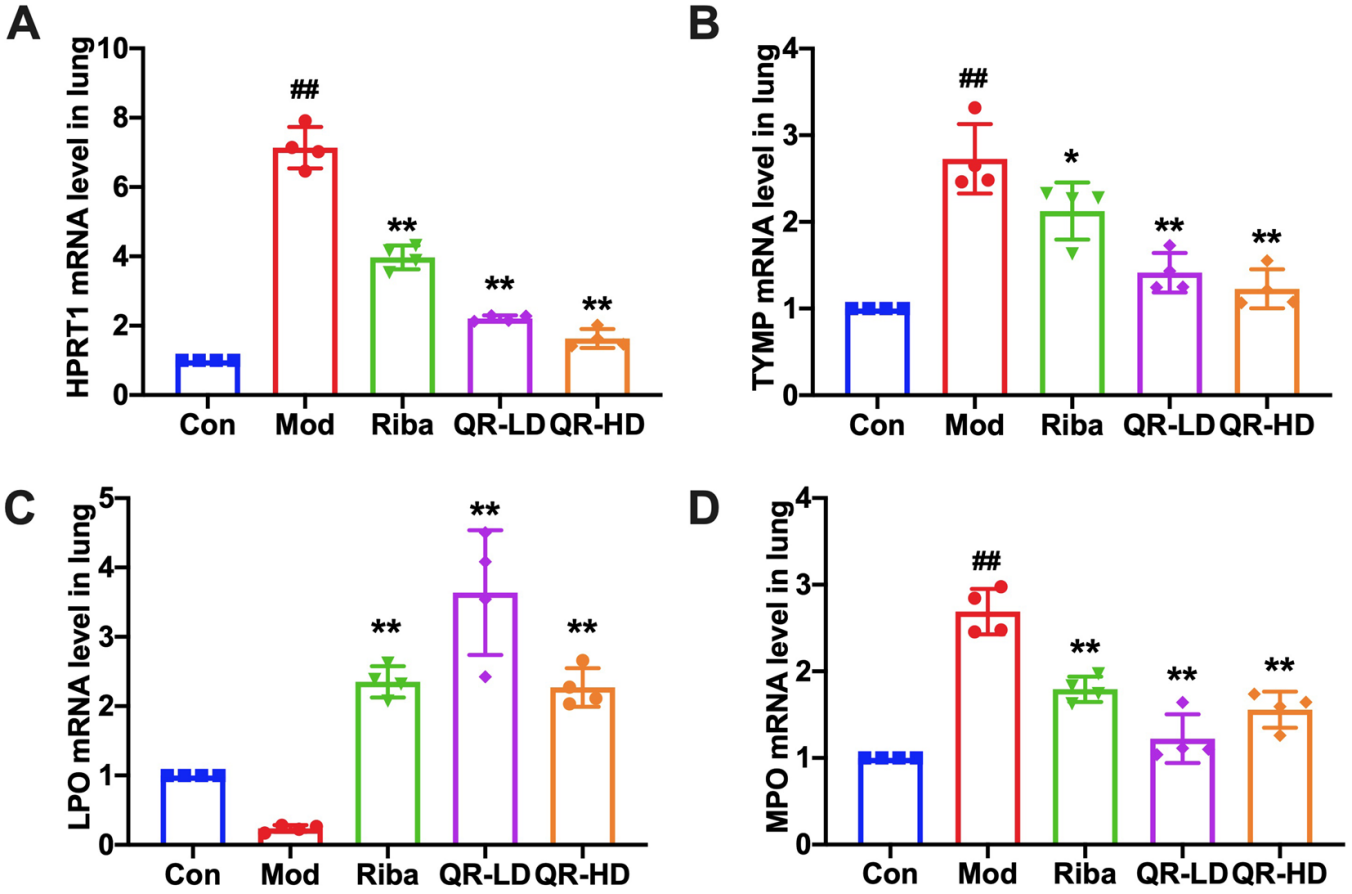
Effects of QR on the key targets. **(A–D)** mRNA Levels of HPRT1, TYMP, LPO, and MPO in lung tissue. Data are presented as the mean ± standard error. Significance: ## *P* < 0.01 vs control group; # *P* < 0.05 vs control group; ** *P* < 0.01 vs model group; * *P* < 0.05 vs model group.

To further validate the results obtained in metabolomics and network pharmacology, we tested the mRNA levels of the key targets. The results showed that the mRNA levels of HPRT1, TYMP and MPO were significantly increased, whereas the levels of LPO were significantly decreased in the model group compared with the control group. In the QR-treated groups, this trend was reversed (Fig. 11A–D).

## Discussion

Human respiratory syncytial virus (RSV) infection is a leading cause of lower respiratory tract diseases in infants and young children, with pneumonia and bronchiolitis as the main clinical symptoms. The World Health Organization estimated that RSV is responsible for over 33 million new episodes of acute lower respiratory infection in children younger than 5 years^46^. RSV causes significant mortality in the developing world, resulting in an estimated 200,000 annual deaths in young children globally, in addition to major morbidity (33.8 million episodes worldwide annually)^47^. Further, RSV is a leading cause of morbidity and mortality in elderly and immunocompromised individuals^48^. However, effective vaccines are not currently available despite high morbidity. Ribavirin and palivizumab are two antiviral drugs approved by the US Food and Drug Administration for treating severe RSV infection. However, the high risk of toxicity associated with ribavirin and the high cost of palivizumab limit the use of these drugs^49^.

In consideration of these limitations of approved drugs, natural products could be sources of promising therapeutic agents for RSV treatment. This study explored the effect of QR on RSV-induced lung inflammatory injury through in vivo experiments. At the same time, it also explored the mechanism of action of QR in treating RSVP through metabolomics and network pharmacology analyses. These results will provide insights for further research on the treatment of RSVP using QR.

First, our in vivo experimental results showed that RSV infection led to significant weight loss in mice, and histopathological sections showed lung consolidation, alveolar wall thickening, and lymphocyte infiltration in the model group mice. Moreover, the immunofluorescence and PCR results showed that the RSV virus levels in the lung tissue and RSV virus mRNA levels of the model group mice were significantly increased, respectively. However, mice in the QR-treated groups (LD and HD) showed significant improvements in all the above indicators. Specifically, QR was able to attenuate weight loss in infected mice and reduce RSV-induced lung tissue damage. Moreover, compared with the model group, the virus levels and the mRNA levels of *RSV-G* and *RSV-F* of the QR-treated group were significantly decreased. This is particularly important, as studies have shown that virus-induced pathogenesis and disease severity are positively correlated with virus levels^50,51^. In addition, lung inflammation caused by RSV infection is a key risk factor for RSV disease severity^52^. Growing evidence suggests that in addition to direct viral damage, uncontrolled inflammation caused by host immune response disorders can also lead to disease severity^45,53^. We therefore examined the effect of QR on the pro-inflammatory cytokines, IL1β, IL2, IL6, TNF-α, and IFN-γ, in RSV-induced mouse lung tissues. The results showed that the levels of IL1β, IL2, IL6, TNF-α, and IFN-γ in the model group were significantly higher than those in the control group. In the QR-treated groups, the levels of these inflammatory factors were significantly reduced in a dose-dependent manner. The above results show that QR ameliorates RSV-induced lung inflammatory injury in the established mouse model.

An untargeted lung tissue metabolomics analysis revealed 52 differential metabolites were identified. Three key metabolic pathways were found, including purine metabolism, cysteine and methionine metabolism, and pyrimidine metabolism. Seven differential metabolites were found to be enriched in the above metabolic pathways, including adenosine 5'-monophosphate, hypoxanthine, uric acid, *S*-adenosyl-l-methionine, *S*-methyl-5'-thioadenosine, deoxycytidine, and beta-alanine. In addition, through a network pharmacology approach, we identified 126 potential therapeutic targets, the enrichment analysis of which suggested that QR may exert anti-RSV and anti-inflammatory effects in vivo mainly by regulating the lipid and atherosclerosis, Kaposi's sarcoma-associated herpesvirus infection, and the AGE-RAGE signaling pathways. Then, the 52 metabolites were imported into Metscape to build a CREG network to identify more targets. By intersecting 244 targets in the CREG network with 126 targets from the network pharmacology analysis, HPRT1, TYMP, LPO, MPO, and CYP19A1 were identified as the common targets. Based on the results of the KEGG metabolic pathways, purine metabolism was considered to be the key metabolic pathway of QR in RSVP treatment, while HPRT1, TYMP, LPO, and MPO were considered the key targets. The key differential metabolites associated with purine metabolic pathways were identified to be adenosine 5'-monophosphate (AMP), hypoxanthine, and urate (uric acid).

Purine metabolism involves the synthesis and decomposition of purine derivatives in vivo*,* including the de novo biosynthetic and purine salvage pathways, as well as degradation^54^. In the de novo biosynthetic pathway, 5-phosphoribosyl 1-pyrophosphate (PRPP) is acted on by a variety of enzymes to generate inosine monophosphate (IMP), which further produces adenosine 5'-monophosphate (AMP), guanine monophosphate (GMP), adenosine, and inosine. Inosine is further converted to hypoxanthine by purine nucleoside phosphorylase (PNP), and finally, uric acid is formed. In the pathway, PRPP amide transferase is a rate-limiting enzyme for purine synthesis^55^ and is regulated by negative feedback from IMP, AMP, and GMP. When AMP is insufficient, uric acid production will be accelerated^56^. In the purine salvage pathway, hypoxanthine–guanine phosphoribosyltransferase (HPRT) and adenine phosphoribosyltransferase (APRT) recycle hypoxanthine and guanine to generate IMP and GMP, thereby controlling uric acid levels. When these enzymes are deficient, this control is lost and uric acid levels rise^57,58^. Thymidine phosphorylase (TYMP) is an enzyme that can catalyze thymidine as thymine. There is a close connection between the control of the thymidine and the level of uric acid^59,60^. Lactoperoxidase (LPO) is a mammalian peroxidase that can react with urate, slowly catalyzing the oxidation of the compound. In this case, an enzyme-urate complex is probably cleaved into dehydrourate and 5-hydroxyisourate, which may affect host defense and inflammatory response^61^.

Several previous studies have reported that uric acid levels in samples of lung aspirates from ICU hospitalized infants positive for RSV infection showed a significant increase in uric acid levels compared with normal samples from infants without RSV infection^62,63^. In addition, experiments in mice showed that the expression of xanthine oxidase (XO, an inflammatory mediator that catalyzes the oxidation of hypoxanthine to xanthine) was significantly increased in the lungs of mice during RSV infection. The uric acid level in the alveolar lavage fluid of mice was also significantly increased. When RSV and XOI (inhibitor of XO) were administered simultaneously, uric acid levels were lowered and lung injury caused by RSV was significantly ameliorated. The main downstream effects of uric acid are Nod-like receptor protein 3 (NLRP3) inflammasome pathway activation and IL-1 production^62,63^. The NLRP3 inflammasome pathway plays an essential role in the excessive inflammatory responses stimulated by various types of viruses, which are closely related to virus-induced lung inflammatory injury^64,65^. The oxidative stress and inflammatory response induced by NLRP3 may promote myeloperoxidase (MPO) secretion^66^. Taken together, all this evidence indicates that the purine metabolism/uric acid pathway is an important metabolic pathway involved in RSV infection, leading to many harmful pathological immune responses.

In this study, quantitative analysis of key differential metabolites showed that the levels of uric acid were significantly increased while the levels of AMP and hypoxanthine were significantly decreased in the model group compared with the control group. The negative feedback effect of low levels of AMP on purine synthesis is thus weakened, causing an increase in the concentration of the substrate for purine nucleotide synthesis, and promoting purine synthesis due to which uric acid content is increased. Interestingly, the hypoxanthine content in the lung tissue of RSV-infected mice was significantly reduced, which may involve an unknown regulatory mechanism. High uric acid levels can stimulate the production of inflammatory cytokines that cause lung inflammatory injury. Thus, our results suggest that purine metabolism disorders may exist in mice during RSV infection, and that QR can reverse these disorders.

Additionally, molecular docking was used to simulate the binding ability of QR and the key targets (HPRT1, TYMP, LPO and MPO). The results showed that QR could stably bind to these targets. Finally, key targets were selected for experimental verification. The results of the QPCR analysis indicated that QR could reverse the abnormal expression of these key targets. The results further confirmed the reliability of metabolomics and network pharmacology in predicting potential therapeutic targets.

## Conclusion

The present study explored the potential mechanisms of QR for RSV-induced lung inflammatory injury treatment using integrated analyses of metabolomics and network pharmacology. The integrated analyses revealed that QR effectively ameliorated RSV-induced lung inflammatory injury, and four key targets (HPRT1, TYMP, LPO, and MPO), one relevant metabolic pathway (purine metabolism), and three key differential metabolites (AMP, hypoxanthine, and uric acid) may play critical roles in the mechanism of efficacy of QR. To our knowledge, this study is the first to predict the importance of a purine metabolism disorder during RSV infection using metabolomics and network pharmacology. These findings provide new insights into therapeutic strategies for RSVP and reveal useful information on the mechanism of QR.

## Supplementary Information

Supplementary Tables.

## Data Availability

The raw datasets generated on the LC–MS/MS instrument during the current study are available from the corresponding author on reasonable request.
