# Supplementary material for: Integrating metabolomics and network pharmacology to assess the effects of quercetin on lung inflammatory injury induced by human respiratory syncytial virus
Source: Sci Rep. 2023 May 17;13:8051. doi: 10.1038/s41598-023-35272-8 (PMC10192330; doi:10.1038/s41598-023-35272-8)
Supplement: Supplementary file 1 — Supplementary Tables. [file 41598_2023_35272_MOESM1_ESM.doc]

Supplementary Table S1. The specific primer sequences used in RT-PCR assay.

| Gene Name | Forward (5′-3′) | Reverse (5′-3′) |
| --- | --- | --- |
| RSV-G | CGGCAAACCACAAAGTCACA | TTCTTGATCTGGCTTGTTGCA |
| RSV-F | CGAGCCAGAAGAGAACTACCAAGG | TGGCGATTGCAGATCCAACACC |
| IL-1β | TCGCAGCAGCACATCAACAAGAG | AGGTCCACGGGAAAGACACAGG |
| IL-2 | TGAGCAGGATGGAGAATTACAG | CAGAGGTCCAAGTTCATCTTCT |
| IL-6 | CTCCCAACAGACCTGTCTATAC | CCATTGCACAACTCTTTTCTCA |
| TNF-α | CCCTCACACTCAGATCATCTTCT | GCTACGACGTGGGCTACAG |
| IFN-γ | CTTGAAAGACAATCAGGCCATC | CTTGGCAATACTCATGAATGCA |
| HPRT1 | GCAGTCCCAGCGTCGTGATTAG | CGAGCAAGTCTTTCAGTCCTGTCC |
| TYMP | GGGAGTGGGTGCCGAGGTAC | CGTCGCTGCTGACTGCTGAG |
| LPO | TGGAGGCTGGTCAAGGATGGTG | TGGATGGTGTGATTCGGTTGGAAC |
| MPO | GCTCCTTGCCTGCCTCATTGG | TGCCAGTGTTGTCACAGATGATACG |
| GAPDH | TGGCCTTCCGTGTTCCTAC | GAGTTGCTGTTGAAGTCGCA |

Supplementary Table S2. 126 potential therapeutic targets.

| AR | EGF | SELE | CRP | MAPK8 | ALB |
| --- | --- | --- | --- | --- | --- |
| PPARG | CD40LG | VCAM1 | CXCL10 | TREM1 | RAB11A |
| PTGS2 | JUN | CXCL8 | CHUK | PPIA | THRB |
| HSP90AA1 | IL6 | DUOX2 | SPP1 | CCL24 | LYZ |
| PIK3CG | CASP3 | HSPB1 | IGFBP3 | CCNA2 | HMGCR |
| ADRB2 | TP53 | TGFB1 | IGF2 | PLA2G10 | ELANE |
| NOS3 | NFKBIA | IL2 | IRF1 | MAPK14 | RBP4 |
| RELA | CASP8 | THBD | ERBB3 | MIF | MME |
| EGFR | TOP1 | SERPINE1 | GSTM1 | TYMP | SELP |
| AKT1 | SOD1 | IFNG | CYP19A1 | CASP7 | S100A9 |
| VEGFA | PRKCA | ALOX5 | SRC | MMP8 | HPRT1 |
| CCND1 | HIF1A | IL1A | KDR | PTPN1 | CD209 |
| BCL2 | STAT1 | MPO | ABCC1 | RHOA | NR3C1 |
| BCL2L1 | CDK1 | ABCG2 | CSNK2A1 | JAK2 | VDR |
| FOS | ERBB2 | NFE2L2 | ALK | NOS2 | PADI4 |
| CDKN1A | CAV1 | PARP1 | ABCB1 | CCL5 | TEK |
| BAX | MYC | SLC2A4 | ARG1 | TYMS | CASP1 |
| MMP2 | GJA1 | CXCL11 | APP | HRAS | LPO |
| MMP9 | ICAM1 | CXCL2 | MMP12 | ERBB4 | CCR4 |
| MAPK1 | IL1B | CHEK2 | CD38 | MAPKAPK2 | ACP1 |
| IL10 | CCL2 | PPARA | PGR | RNASE3 | CREB1 |

Supplementary Table S3. 244 potential targets in the CREG network.

| GNE | HSD17B12 | ALDH3A2 | AKR1D1 | CYP2D6 | UGT1A8 | HPRT1 | GNPTAB |
| --- | --- | --- | --- | --- | --- | --- | --- |
| IL18BP | PIGP | NT5C2 | SRM | CYP2E1 | UGT1A7 | HSD3B1 | HSD17B8 |
| LYPLA1 | NT5C3 | MYCBP2 | SUV39H1 | CYP2F1 | UGT1A6 | HSD3B2 | SRD5A3 |
| UBE2E3 | PDE1A | ICMT | TAF9 | CYP2J2 | UGT1A5 | HSD11B1 | SUV39H2 |
| CARM1 | PDE1C | CLCF1 | TPMT | CYP3A4 | UGT1A9 | HSD11B2 | UGT2A3 |
| UGT2B11 | PDE2A | LYPLA3 | TPO | CYP3A5 | UGT1A4 | HSD17B1 | EHMT1 |
| PDE10A | PDE3A | GAD1 | UBA1 | CYP4B1 | UGT1A1 | HSD17B3 | SETD7 |
| EHMT2 | PDE3B | GAD2 | UBE2A | C9orf98 | UGT1A3 | HSD17B2 | C1orf25 |
| UGT2A1 | PDE4A | CYP4X1 | UBE2B | CYP11B1 | WHSC1L1 | APRT | EPX |
| UBE2C | PDE4B | AMD1 | UBE2D1 | CYP11B2 | UBE2R2 | ENTPD8 | ADPGK |
| SAT2 | PDE4C | AK5 | UBE2D2 | CYP19A1 | UBE2W | SETD8 | SETDB2 |
| CYP4F8 | PDE4D | AMDP1 | UBE2D3 | CYP21A2 | UBE2Q1 | UBE2NL | DOT1L |
| LYPLA2 | UBE2J1 | GCNT1 | UBE2E1 | DCK | TRMT1 | TPMTP1 | NT5C1A |
| CLC | PDE6D | AMPD1 | UBE2E2 | UGT3A2 | TRMU | LPO | CNDP1 |
| UBE2J2 | HSD17B7 | AMPD2 | UBE2G1 | DNMT1 | ASH1L | APOBEC4 | PDE8B |
| AK7 | PDE6G | PDE7B | UBE2G2 | TRDMT1 | METTL3 | MAT1A | AKR1C3 |
| CANT1 | PDE6H | AMPD3 | UBE2H | DNMT3A | NT5M | MAT2A | PDE5A |
| ADK | PDE7A | UBE2S | UBE2I | DNMT3B | AICDA | METTL1 | RNMT |
| UBE2U | PDE8A | MAT2B | UBE2L3 | ABAT | AS3MT | MPO | UBE2M |
| CYP1A1 | PDE9A | UBE2E4P | UBE2N | TYMP | CYP4F11 | ASMT | PIGQ |
| CYP1A2 | PDE1B | SETD2 | UBE3A | B3GNT6 | MLL3 | MTAP | GCNT3 |
| CYP1B1 | DPH5 | UBE2T | UGT2B@ | CYP4Z1 | SAT1 | MTRR | UBE2L6 |
| CYP2A6 | UBE2D4 | N6AMT1 | UGT2B4 | AK1 | NSD1 | NP | UBE2Q2 |
| CYP2A7 | UPB1 | LGALS13 | UGT2B7 | AK2 | SMYD3 | NT3 | NT5C1B |
| CYP3A7 | PIGA | CYP2S1 | UGT2B10 | AK3L1 | CYP3A43 | NRF1 | ENTPD1 |
| CYP2A13 | PIGC | HAS1 | UGT2B15 | ALDH2 | UBE2Z | NT5E | ENTPD3 |
| CYP2B6 | PIGH | HAS2 | UGT2B17 | ALDH3A1 | CYP4F12 | ALDH7A1 | PRDX6 |
| CYP2C8 | PLA2G4A | HAS3 | NULL | ALDH1B1 | SMS | PAM | SETD1A |
| CYP2C9 | UGT2B28 | NT5C | WHSC1 | ALDH1A3 | UAP1 | RDH8 | CDA |
| CYP2C18 | UGT1A10 | UBE2K | XDH | C6orf199 | SRD5A1 | PDE11A | SETDB1 |
| ALDH9A1 | SRD5A2 | PCMT1 | CDC34 |  |  |  |  |
